# Supplementary material for: Competing transfer pathways in direct and indirect dynamic nuclear polarization magic anglespinning nuclear magnetic resonance experiments on HIV-1 capsid assemblies: implications for sensitivity and resolution
Source: Magn Reson (Gott). 2021 Apr 27;2(1):239–49. doi: 10.5194/mr-2-239-2021 (PMC8203495; doi:10.5194/mr-2-239-2021)
Supplement: Buildup profile for 13C signals in DNP-enhanced CPMAS spectra of tubular assemblies of 5F-Trp, U-13C, 15N CA containing 22.8 mM AMUPol. The supplement related to this article is available online at: https://doi.org/10.5194/mr-2-239-2021-supplement. [file mr-2-239-supplement.pdf]

Supplement of Magn. Reson., 2, 239–249, 2021
https://doi.org/10.5194/mr-2-239-2021-supplement
© Author(s) 2021. CC BY 4.0 License.

Open Access
MAGNETIC RESONANCE

Supplement of

Competing transfer pathways in direct and indirect dynamic nuclear polarization magic anglespinning nuclear magnetic resonance experiments on HIV-1 capsid assemblies: implications for sensitivity and resolution

Ivan V. Sergeyev et al.

Correspondence to: Tatyana Polenova (tpolenov@udel.edu) and Angela M. Gronenborn (amg100@pitt.edu)

The copyright of individual parts of the supplement might differ from the article licence.

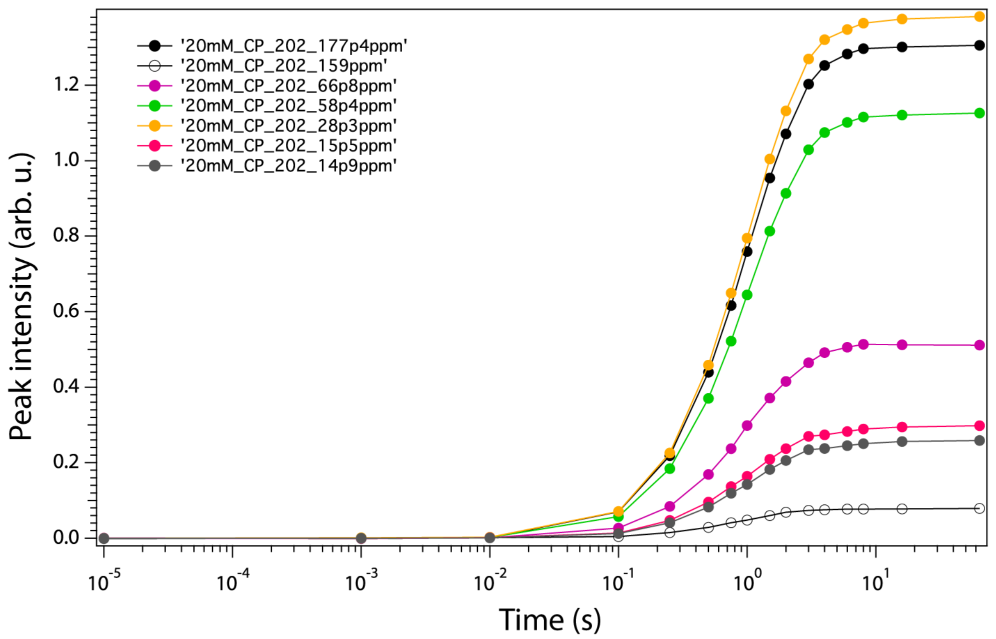

Figure S1. Buildup profiles for ^{13}C signals in DNP-enhanced CPMAS spectra of tubular assemblies of 5F-Trp,U-^{13}C,^{15}N CA containing 22.8 mM AMUPol. Signals corresponding to different functional groups are color coded and the corresponding chemical shifts are displayed on the bottom right. The spectra were acquired at 14.1 T (150.96 MHz ^{13}C Larmor frequency) at MAS frequency of 24 kHz and temperature of 120 K.
